# Soluble Major Histocompatibility Complex I-Related Chain A (sMICA)*008 Levels Associate with Smoking, Presence of Chronic Obstructive Pulmonary Disease, and Prevalence of Lung Cancer

**DOI:** 10.3390/jcm15041496

**Published:** 2026-02-14

**Authors:** Robert M. Burkes, Mauricio Orozco-Levi, Alba Ramirez-Sarmiento, Albert Sanchez-Font, Joaquin Gea, Michael T. Borchers

**Affiliations:** 1 Division of Pulmonary, Critical Care and Sleep Medicine, Department of Internal Medicine, College of Medicine, University of Cincinnati, Cincinnati, OH 45267, USA; 2 Department of Veterans Affairs, VA—Cincinnati Medical Center, Cincinnati, OH 45220, USA; 3 Research Center, Fundación Cardiovascular de Colombia, Calle 158 A # 23-58, Floridablanca 681004, Santander, Colombia; 4 Group of Research in Muscle, Training and Lung Diseases (EMICON), Ministry of Science and Technology (MINCIENCIAS), Calle 26 # 57-83, Bogotá 111321, Colombia; 5 Respiratory Department, Hospital Internacional de Colombia, Fundación Cardiovascular de Colombia, Kilómetro 7 Autopista Piedecuesta, Floridablanca 681004, Santander, Colombia; 6 Department of Medicine, Universidad de Santander (UDES), Calle 70 # 55-210, Bucaramanga 680006, Santander, Colombia; 7 Respiratory Medicine Department, Hospital del Mar, Passeig Maritim 25, 08003 Barcelona, Spain; 8 Department of Medicine and Life Sciences, Universitat Pompeu Fabra (UPF), 08002 Barcelona, Spain; 9 Instituto Municipal de Investigación Médica (IMIM), 08003 Barcelona, Spain

**Keywords:** smoking, COPD, lung cancer, NK cells, innate immunity

## Abstract

**Background:** Lung cancer and chronic obstructive pulmonary disease (COPD) are morbid and mortal conditions arising from noxious endothelial stress. Soluble Major Histocompatibility Complex I Chain Related A (sMICA) is an activating ligand for the NKG2C receptor, and the soluble form indicates endothelial stress and is a mechanism for evading immune surveillance in lung cancer. We provide independent associations between sMICA*008 levels and the prevalence of lung cancer, lung cancer histologies, COPD, and risk factors for both diseases. **Methods:** We describe statistical associations between sMICA and demographic and clinical variables. Multivariate linear regression determined the independent associations between sMICA levels and lung cancer histology, between those with and without primary lung cancer, and prevalent COPD in participants without lung cancer. Point estimates and 95% confidence intervals are reported; *p* < 0.05 is considered statistically significant. **Results:** The cohort (*n* = 586 patients) included 24% female and 48% current or former smokers. Mean sMICA were 5.20 pg/mL ×10^2^, and FEV1%-predicted of 62. sMICA levels were higher in those who smoked vs. those who did not. In Multivariate regression, non-small cell lung cancer (NSCLC) was associated with 14.2 pg/mL ×10^2^ (95% CI 3.57 to 24.9 pg/mL ×10^2^) higher sMICA levels compared to those without cancer. No other histology was independently associated with higher sMICA. Primary lung cancer [12.5 pg/mL ×10^2^ (2.85 to 22.2 pg/mL ×10^2^)] and COPD in those without cancer [4.38 pg/mL ×10^2^ (0.38 to 8.39 pg/mL ×10^2^)] were associated with higher sMICA. **Conclusions:** sMICA*008 is independently associated with NSCLC, primary lung cancer, and COPD, respectively, in a cohort of current, former, and never smokers with and without lung cancer. sMICA levels were also higher in smokers. This study provides a foundation for future studies on sMICA activity in lung cancer and COPD, and assessment of sMICA as a biomarker for lung cancer cell type and risk of lung function loss in COPD.

## 1. Introduction

Lung cancer and chronic obstructive pulmonary disease (COPD) are highly mortal diseases driven by personal exposure to combustible tobacco or biomass smoke [1,2]. Their relationship is likely synergistic, with COPD being associated with worse clinical lung cancer outcomes [3]. Noxious exposures promote inflammatory stress on airways, leading to altered epithelial and cellular biology that may drive both COPD and lung cancer. Of particular interest is Major Histocompatibility Complex I-related Chain A (MICA), a stimulating ligand for the NKG2D activating receptor found on cytotoxic lymphocytes (CTLs) such as natural killer (NK) cells and CD8 T cells. MICA expression is increased in airway epithelial cells under stress and may not normalize once an insulting agent is removed [4,5]. Increased inflammatory potential of NK cells has been shown to be directly toxic to lung tissue in vitro [6]. Chronic noxious stimulation further leads to MICA being cleaved from cellular surfaces (including epithelial cells and endothelial cells), becoming soluble (sMICA) [7,8]. sMICA, in turn, acts as a ligand for NKG2D receptors found on CTLs [9]. Shedding of usually membrane-bound MICA into the peripheral blood is a potent mechanism by which malignant cells evade immunosurveillance, allowing for tumor growth and metastasis [9,10]. sMICA cleaved from the surface of stressed, infected, or damaged cells binds to NKG2D receptors on CTLs and effectively lowers the surface expression of NKG2D, inhibiting the ability of CTLs to activate and eliminate malignant threats [10]. While increased membrane-bound MICA on epithelium is seen in COPD [11], stressed cells and sMICA allow tumors to evade immune surveillance [12]. While these are two separate aspects of MICA biology, the potential for sMICA to act as an autoantigen for NK cells [7] suggests sMICA levels may play a role in both the development of COPD as well as lung cancer. We present a cross-sectional analysis of 5 participants with smoking, spirometry, comorbidity, drug treatment, and malignancy data, wherein we determine statistical associations between peripheral levels of soluble MICA*008 (hereafter referred to as sMICA) and lung cancer cell type and prevalent airflow limitation. As the MICA ligand is highly variable, and despite our study assessing the most common ligand (MICA*008) [13], we will perform a subset analysis of those with detectable sMICA levels to determine if associations remain when accounting for only those participants who express MICA*008.

The clinical approach to lung cancer has become increasingly biomarker-based as therapy and prognosis have been connected to the expression of certain genetic and molecular markers [1,14,15,16]. Discovery of blood-based biomarkers able to predict lung cancer outcomes remains elusive despite several markers, found in low serum concentration, having the potential to be leveraged for this action [14]. Recently, machine-learning models using biomarkers found in low serum levels have had some early success in delineating lung cancer presence and biology [17], but this work is in its infancy. While exposures, most notably combustible tobacco products [18], are well-known and well-defined risk factors for lung cancer, the presence and pattern of emphysema may also be associated with lung cancer risk [19]. Describing unifying pathobiological mechanisms that underlie the connection between smoking, resultant lung parenchymal destruction, and malignant conversion will be useful in understanding shared mechanisms of COPD and lung cancer. The mechanisms of lung function loss in COPD, as well as markers integral to lung cancer biology, are important for future investigation. Our study provides foundational evidence for further assessment of the role of sMICA in the pathological mechanisms of COPD and lung cancer, and increasing the risk of these conditions.

In this manuscript, we describe independent statistical associations between sMICA concentration, smoking, airflow limitation, and the presence of lung cancer. We analyzed a large population of participants with well-defined historical and clinical data, including smoking-related lung disease data. We compare sMICA levels among lung cancer types, lung cancer and cancer in other organs, and in COPD vs. not in those without lung cancer. We provide multilevel models accounting for clinically and biologically relevant covariates to determine independent associations. Due to the variability of MICA in humans, we expected many participants not to have detectable MICA*008 and to perform a subset analysis excluding those with absent sMICA*008. We hypothesized that sMICA levels would be higher in those with cancer and airflow limitation, with a potential for differential levels among particular lung cancer types.

## 2. Methods

### 2.1. Study Participants

The Research Committee of Human Investigation at the Municipal Institute for Medical Research (IMIM) approved the study (Ref. ID2005/2119/I; 9 February 2009), and written informed consent was obtained from each participant after a full explanation of the purposes and characteristics of the study. The research was designed as a cross-sectional study according to the guidelines for human research [20].

This study investigates a clinical database of 586 participants seen over a period of 10 contiguous years at Parc de Salut Mar (Barcelona, Spain) as part of a study program (FIS Health Research Fund grants, codes FIS PI08/1612), which proposed to investigate MICA activity in response to tobacco, susceptibility to COPD, and possible associations with lung cancer. This database included current and former smokers along with non-smoking controls with and without COPD (defined as a history of smoking, symptoms congruent with COPD, and Global Initiative for Chronic Obstructive Lung Disease-defined airways obstruction [21]) who were recruited from the outpatient clinics at Parc Salut del Mar Hospital. Those with tissue biosamples from endoscopic lung biopsy had these results included. Further, blood sMICA sampling was performed, and accompanying demographic, clinical, spirometric, imaging, and tumor histology data were collected. Participants in the COPD arm were considered clinically stable without an exacerbation of COPD (AECOPD) within two months prior to enrollment. Along with COPD patients who were not considered “stable”, those with clinical suspicion of bronchial asthma or AECOPD treated with systemic steroids within the two months preceding enrollment were excluded, as were patients who met the above inclusion criteria but elected not have their data collected.

Demographic data (age and sex) were collected at enrollment. Smoking status was collected via questionnaire at enrollment, and participants were defined as never smokers (<100 cigarettes smoked over a lifetime), former smokers, and current smokers if they were actively using combustible tobacco within the year prior to enrollment. Smoking history was quantified as pack-years smoked. Pulmonary function testing was performed by American Thoracic Society/European Respiratory Society guidelines [22], and absolute and %-predicted values are reported (normalized for a Mediterranean population). The presence of COPD (defined as having a forced-expiraotry-volume-in-1 s/forced vital capacity ratio < 0.70) and disease severity were determined based on GOLD criteria [21]. All participants not meeting this spirometry cutoff were labeled as having “preserved” lung function. Participants were asked to report statin use at the time of enrollment. The presence of lung cancer (or a primary tumor in another system) at enrollment was recorded, and the histology of tumors was abstracted from participant records. Cancer type is defined as no cancer vs. non-small cell lung cancers (which include adenocarcinomas, squamous cell carcinomas, and poorly differentiated carcinomas), small cell lung cancer, and malignancies that are not lung-primary. Cancer was also modeled as lung primary (which includes NSCLC and SCLC) vs. primary malignancies in other systems, and no cancer. Stage for non-small cell lung cancers was described using the TMN lung cancer staging guidelines [23].

### 2.2. Detection of Circulating sMICA

Peripheral venous blood samples (5 mL in a vacutainer without anticoagulant) were obtained by needle puncture of the basilic or cephalic vein of the non-dominant arm. The samples were kept on ice for transport and centrifuged at 4 °C, 2500 rpm for 15 min. Serum fractions were stored in 500 microliter aliquots at -70ºC until further processing. The sMICA*008 levels were determined using the ELISA “sandwich” technique [24]. The anti-MICA capture antibody AMO1 (5 µg/mL) was diluted in PBS. BSA (15%) was used to block nonspecific binding sites. Recombinant sMICA*004 protein was included as a positive control. For detection, the respective serum samples (1:3 dilution in 7.5% BSA) and the positive control were incubated with the anti-MICA BAMO3 antibody at concentrations of 1µg/mL. Anti-IgG2a-HRP (1:8000 dilution; Southern Biotechnologies, Birmingham, AL, USA). The plates were washed and developed using the TMB Peroxidase Substrate System (KPL, Gaithersburg, MD, USA). The absorbance was measured at 450 nm. All samples were processed in triplicate in batches. The sMICA assay limit of detection is 20 pg/mL, inter-assay correlation of variance is 6.6%, and intra-assay correlation of variance is 5.1%.

### 2.3. Study Design

Descriptive statistics were used to examine differences in demographic and clinical factors among the study population stratified, respectively, between cancer type and sMICA levels. Chi-squared testing was used to determine differences between categorical variables, while Student’s *t*-test or analysis of variance (ANOVA), depending on the number of observations, is employed to describe the differences in continuous variables across categorical classifications.

Bivariate and Multivariate linear regression modeling was used to determine statistical associations between lung cancer type (exposure) and sMICA (outcome) levels. An indicator variable of ‘no cancer’ was used, and the results were given as the comparison of each cancer type with the indicator variable. Multivariate models included the clinically relevant covariates [25] of %-predicted FEV1 (which includes terms for FEV1, age, sex, race, and height, obviating the need to control for these variables, which otherwise introduces collinearity), smoking status (current vs. former vs. never as the reference value), and statin therapy (which has been shown to directly alter membrane-bound MICA expression [26]). Models were also repeated with the presence of primary lung cancer vs. non-primary lung cancer. We assessed the sMICA difference (when controlled for smoking status and statin therapy) in those with COPD vs. preserved lung function in a subset without lung cancer, to determine an independent association between circulating sMICA levels and reduced lung function.

To assess the associations between increasing sMICA on odds of having prevalent lung cancer, we have used multinomial logistic regression with a per-100-pg/mL increase in sMICA as the main exposure and outcomes of NSCLC, SCLC, large cell, or non-lung primary tumors vs. no cancer, again, as the indicator variable. Multivariate multinomial logistic regression analyses used the same covariates as above. Like above, the models were also assessed with primary lung cancer vs. not as the main outcome.

The results of linear regression modeling report sMICA point estimates with 95% confidence intervals, while logistic regression reports odds ratios with 95% confidence intervals. For all analyses, *p* < 0.05 is considered statistically significant. Missingness is infrequent and is considered to occur at random. Statistical analyses were performed using STATA version 15.1 (College Station, TX, USA).

A sensitivity analysis was performed, including only those that had sMICA values greater than the lower limit of detection.

## 3. Results

### 3.1. Description of Study Population

A total of 567 participants were studied (Table 1). The median age is 67 years old, and 24% are female. Across the population, 58% of participants had ever smoked, and 29% reported smoking at the time of enrollment. The average %-predicted FEV_1_ was 62%, and the transfer factor for the diffusion of carbon monoxide/diffusion capacity (DLco) was 73% of predicted. Statins were prescribed to 20% of the study population, and the average sMICA concentration was 5.20 × 10^2^ pg/mL.

Across cancer groups (Table 1), those with primary lung cancer were more likely to have ever smoked and to be current smokers (*p* < 0.001 for both). There was a statistically significant difference across each lung cancer type when lung function was measured in volume (p = 0.02), but this association was not seen when the corrected %-predicted FEV1 value was used (*p* = 0.10). Diffusion capacity (DLco) was different across lung cancer types (*p* < 0.001), with small cell having the lowest measured DLco, numerically. Across cancer groups, participants with lung cancer had higher sMICA readings than those without cancer or primary tumors outside of the pulmonary system (*p* < 0.001).

The association between sMICA levels and other clinical parameters is described in Table 2. There was no difference in sMICA levels when participants were categorized as never smokers, former smokers, and current smokers. However, when dichotomized as ever vs. never smokers, ever smokers have a statistically significantly higher sMICA level than never smokers (*p* = 0.04). Mean sMICA levels were higher in those with primary lung cancer (15.9 × 10^2^ pg/mL) vs. those without (2.31 × 10^2^ pg/mL) primary lung cancer (*p* < 0.001). Further, there was a statistically significant difference in sMICA levels between those currently not smoking (1.77 × 10^2^ pg/mL), current smokers without lung cancer (3.11 × 10^2^ pg/mL), and those with lung cancer (14.9 × 102 pg/mL) (*p* < 0.001). sMICA levels were not statistically significantly different among statin users, GOLD stage, or cancer stage.

### 3.2. sMICA and Cancer Types

When broken down by cancer type (NSCLC, SCLC, Large Cell, and cancers of other organs) and compared to those without cancer, those with NSCL had on average 13.2 × 10^2^ pg/mL higher sMICA levels (95% CI 7.39 to 19.1 × 10^2^ pg/mL, *p* < 0.001) in bivariate analysis. Neither SCLC, large cell lung cancer, nor cancers of other organ systems had statistically significantly different sMICA levels from those without cancer in bivariate analysis. In Multivariate analysis, when controlled for clinically relevant covariates, NSCLC was associated with a 14.1 × 10^2^ pg/mL higher MICA level (95% CI 3.42 to 24.8 × 10^2^ pg/mL, *p* = 0.01) when compared to those without cancer (Table 3). The other cancer types were not associated with higher sMICA levels when compared to those without cancer in the Multivariate analysis.

Those with primary lung cancer of all types were compared to those without primary lung cancer. In bivariate analysis, primary lung cancer was associated with a 12.7 × 10^2^ pg/mL higher sMICA than those who did not have primary lung cancer (95% CI 7.39 to 19.1 × 10^2^ pg/mL, *p* < 0.001). In Multivariate regression analysis controlled for clinically relevant covariates (Table 4), primary lung cancer was associated with a 12.4 × 10^2^ pg/mL higher sMICA than those without primary lung cancer (95% CI 2.85 to 22.0 × 10^2^ pg/mL; *p* = 0.011).

Participants with lung cancer were compared to smokers without lung cancer. Using the same covariates as above, those with primary lung cancer had a statistically significant 11.2 × 10^2^ pg/mL (0.28 to 22.1 × 10^2^ pg/mL; *p* = 0.02) higher sMICA levels compared to smokers without lung cancer.

We used multinomial logistic regression analysis to describe the increase in odds of particular types of lung cancers with increased MICA when compared to those without cancer. In bivariate analysis, every 100 pg/mL increase in MICA was associated with a 2.0% increase in odds of NSCLC (95% CI 1.01–1.03, *p* = 0.005) when compared to a similar increase in those without cancer. No other cancer types (SCLC, cancer of other organ systems) were associated with a similar increase in odds of prevalence with increasing MICA levels when compared to those without cancer. In Multivariate analysis, when controlled for clinically relevant covariables, NSCLC also had a 1.6% increase in odds of prevalence (95% CI 1.0009–1.03, *p* = 0.046; *p* = 0.038) per 100 increase in MICA when compared to those without cancer. As with the above, there was no association between MICA increase and increased odds of SCLC or cancer in other organs when compared to those with no cancer.

When comparing any primary lung cancer to those without lung cancer, in bivariate analysis, every 100 pg/mL increase in MICA is associated with 2.1% greater odds of primary lung cancer (95% CI 1.01–1.03, *p* = 0.002). In Multivariate analysis, every 100 unit increase in MICA was associated with a 1.3% increase in odds (95% IC 1.0006–1.03, *p* = 0.04) of primary lung cancer.

### 3.3. sMICA and COPD

Expanding upon previous findings in which COPD patients were found to express more MICA on epithelial cells than those without COPD, we assessed participants without cancer to see if there was a statistically significant difference in concentrations of sMICA when controlled for factors thought to impact sMICA levels. To examine statistical associations between sMICA levels and airflow limitation, we compared average sMICA levels among COPD vs. those without COPD in a subset without lung cancer. In bivariate analysis, including only those without cancer, participants with COPD had an average of 3.91 × 10^2^ pg/mL higher sMICA in the serum (95% CI 1.47 to 6.36 × 10^2^ pg/mL; *p* = 0.002). In Multivariate linear regression, when controlling for smoking status and statin use, those with COPD had a statistically significantly higher sMICA level than those with preserved lung function [4.38 × 10^2^ pg/mL (95% CI 0.38 to 8.39 × 10^2^ pg/mL); *p* = 0.03] (Table 5).

### 3.4. Sensitivity Analysis

Due to the large number of participants without measurable values, we performed a sensitivity analysis assessing only those participants with detectable sMICA*008 levels (*n* = 191; Supplementary Tables S1–S4). In this analysis, sMICA levels remained higher in primary lung cancer vs. the rest of the study population, as well as in those with lung cancer vs. smokers without cancer and non-smokers. In this sensitivity analysis, NSCLC was independently associated with higher MICA levels 53.6 × 10^2^ pg/mL [(95% CI 26.9 to 80.3 × 10^2^ pg/mL); *p* < 0.001] when compared to those without lung cancer. Similarly to the entire study population, a statistical association was not seen with SCLC or cancer in other organs. In those without lung cancer, sMICA remained independently associated with COPD: [32.2 × 10^2^ pg/mL (95% CI 15.9 to 48.6 × 10^2^ pg/mL); *p* < 0.001].

## 4. Discussion

In a study population of 586 participants, non-small cell lung cancer (NSCLC) is independently associated with higher sMICA levels when compared to those without lung cancer, when controlled for clinically relevant covariables. These associations remained when participants without detectable sMICA*008 were excluded from analysis. Additionally, in participants without lung cancer, COPD was independently associated with higher sMICA levels. The difference was smaller in the COPD analysis, likely due to less cleavage of MICA from epithelium in COPD, or that the presence of a malignancy is a particularly potent cause of membrane-bound MICA cleavage. These characterizations suggest sMICA may be a mediator of malignant transformation, cancer cell survival, and lung function loss, and inform further studies into the interface of sMICA and cytotoxic lymphocyte activity as it relates to lung cancer and COPD, respectively.

Levels of MICA in serum, on cell surfaces, and in cellular cytoplasm and clinical outcomes have been described in multiple types of cancers in a meta-analysis [27]. This study did not report outcomes associated with sMICA in lung cancer participants. One study of 207 participants recruited over eight years suggests that higher sMICA levels are associated with more advanced lung cancer and lower survival rates [28]. The authors, however, were not able to incorporate lung function, an important clinically relevant factor in the prognostication of NSCLC survival [29]. In other malignancies, such as breast cancer, sMICA levels were found to be higher in those with well-differentiated breast cancers than in those with poorly differentiated malignancies [30]. Similarly to our analysis of sMICA levels and odds of lung cancer, higher sMICA levels were associated with increased odds of pancreatic cancer [31]. We expand previous lung cancer findings by modeling for lung function and by demonstrating that elevated sMICA levels are associated with the presence of NSCLC and not with other forms of lung cancer or cancers in other solid organs metastatic to lung compared to participants without cancer, independent of lung function, smoking status, and statin (which have been shown to alter expression of membrane-bound MICA which could conceivably influence levels of the soluble product [26]) use. It can be postulated that the differential levels across cancer types are due to the epithelial origin of NSCLC. Our findings supplement previous literature by showing that sMICA levels are specifically elevated in NSCLC when controlled for clinically relevant covariates. Levels in our study are also higher than those in studies of other malignancies. One reason may be the influence of field cancerization over the broad surface area of the lungs, which is described to be particularly pertinent to NSCLC [32,33]. Other hypotheses include the amount of MICA expression in the pulmonary compartment, as well as the main risk factor for lung cancer being direct exposure to noxious irritants, such as cigarette smoking, promoting a more robust MICA cleavage. While our findings are foundational, these may suggest that the role of sMICA in immune system evasion may be more relevant and potentially targetable in NSCLC.

With further analysis focused on the predictive value of sMICA levels in a clinical setting, plasma sMICA levels may provide a complementary prognostic tool in cancer to supplement current biomarkers, which identify cancer cell types [15]. Current histology-specific biomarkers support those that involve genetic analysis of cancer cells and leverage the identification of specific genes to inform prognosis and therapy [15,34]. While sMICA is thought to increase in late-stage tumors of multiple primary organs [35] our analysis suggests that in those with a lung tumor, sMICA may best identify those with a primary lung malignancy, specifically NSCLC. Using sMICA in the diagnostic pathway may be useful as an early decision-making and prognostic tool and as a predictive marker of a lung primary tumor. This potential is supported in other studies showing that those with elevated sMICA in hepatocellular carcinoma have larger tumors and earlier mortality than those without elevated sMICA [36]. While feasible, limitations exist, as demonstrated by our study, in which many of the participants did not have detectable sMICA*008. While this is the most common MICA allele, variation makes broad generalization difficult. Further translational work in successfully implementing sMICA and other soluble NKG2D ligand levels into prognostic algorithms is necessary.

Our study describes higher sMICA levels in those with COPD than those without when controlled for clinically relevant covariables. The source of sMICA in those without a malignancy is less clear, but may be related to a combination of injured airways epithelium and endothelium that share in environmental stressors thought to drive COPD and the development of lung cancer [37]. A peripheral source for sMICA is feasible, as endocytosis of endothelial-bound MICA and subsequent release into plasma may not fully account for increased sMICA levels in those without cancer. Our study further suggests that controlling for active smoking does not attenuate the statistical association between sMICA and reduced lung function, suggesting that active smoking is not the sole cause of elevated sMICA levels in COPD. We cannot define the variation in sMICA levels over time, but elucidating the trend of sMICA over time in COPD and the impact on CTL and NK cell biology may provide insight into mechanisms of disease development.

There is a paucity of data regarding a potential biological role of MICA in COPD. Available literature suggests that MICA is presented by epithelial cells in COPD patients [11] and may lead to altered NK cell functioning, promoting the presence of opportunistic infection [38]. Previous studies assessing the stimulation of the NKG2D receptor on cytotoxic lymphocytes in the lung suggest that epithelial expression of MICA is not differentially based on COPD status [39], but is increased in smokers [40], which would be expected due to increased epithelial stress. While we control for combustible tobacco use and find that there are statistically significantly higher sMICA levels in COPD when adjusted for smoking status, the effect is not as large as is seen in NSCLC. We believe the higher levels of sMICA may be due to sustained epithelial stress from the multifactorial chronic inflammatory milieu of COPD, and increased sMICA can be considered to be present in a similar fashion to the so-called ‘alarmins’ or ‘damage-associated molecular patterns’, which are also found in higher concentrations in COPD compared to those without obstruction [41,42]. Of note, in the entire study population, there was no association between sMICA levels and GOLD stage, and lower lung function did not attenuate the statistical association between sMICA levels and NSCLC. We would suggest that, while MICA expression increases due to multiple means of biologic stress, malignant transformation may be a more potent inducer of MICA cleavage. This is to say, the importance of ongoing multifactorial immune-and-exposure-driven irritation (i.e., chronic infection, propensity for active infection, environmental factors, poor nutrition impacting immune activity, and ongoing smoking) may be more important to the identification of high circulating levels of sMICA than the clinical stage of COPD. With a paucity of previous studies, our findings for COPD, specifically, remain theoretical. Despite this, cytotoxic lymphocytes expressing the NKG2D receptor likely play an active role in the hyperinflammatory status of COPD [43], and soluble ligands that increase in COPD and directly impact lymphocyte function are worthy of further investigation. As cytotoxic lymphocyte- and NKG2D-targeted therapies are possible, the effect of high levels of sMICA on immunologic pathways in COPD could prove to be beneficial in the future.

While personal tobacco smoking is the main risk factor for COPD, not all smokers develop the disease [44]. Mechanisms leading to abnormal immune response and markers of these overactive responses are needed. sMICA may be a marker of poor outcomes in COPD deserving of further study. Studies on lung transplant patients suggest that elevated anti-MICA antibody titers may induce a pro-inflammatory response that contributes to graft rejection [45]. Although speculative, MICA molecules can function as alloantigens [46] and may feed forward into a pro-inflammatory cycle leading to parenchymal destruction. At the time of this writing, there is equipoise surrounding the patient-directed anti-inflammatory medications (e.g., inhaled corticosteroids and chronic macrolide use) in COPD therapeutic pathways. As inflammation in COPD persists to some degree despite smoking cessation [47], markers such as sMICA may be corollaries for ongoing inflammation and targets for patient-directed therapies. Further, sMICA levels could be a source of ongoing inflammation, as suggested by the similarities in levels between former and current smokers; however, the strength of this statistical association remains speculative.

A challenge going forward, as noted by our large confidence intervals in a relatively large study population, will be calibrating sMICA levels to be clinically meaningful. The most utilitarian means of calibrating biomarkers is of ongoing interest [48]. Means of calibration across multiple sample batches have been reported, which can be leveraged in future sMICA studies [49]. In the future, translating sMICA levels for use as targets in clinical trials or for clinical use will require large cohorts of participants with well-defined clinical variables to find thresholds at which the circulating levels of sMICA are thought to associate with a higher risk of poor outcomes.

This study has several limitations. Importantly, the ELISA assay only detects the MICA*008 subtype, which is the most common subtype of MICA. As such, total sMICA levels may be underestimated. While we provide sensitivity analysis of only those with measurable sMICA*008, the large number of participants without detectable levels makes drawing a global conclusion of all sMICA subtypes as a biomarker difficult, and suggests further study and refinement of using sMICA to detect clinical outcomes would be needed. However, we can abstract clinical pathology results to ensure the accuracy of our reported lung cancer cell types. Also, this is a cross-sectional analysis that relies on retrospective chart review and cross-sectional study and, as such, cannot imply causality. This study was conducted in a single center, which limits generalizability. The study does not account for all possible residual confounding of marker levels. Variables collected by participant recall (smoking status and history) are incomplete in certain instances, potentially introducing bias, and these variables are also potentially impacted by recall bias. Further, we do not have a report of pack-years smoked in the cohort, which would provide a covariable that would greatly strengthen our approach. Moreover, our analysis of statin use could not verify doses or adherence to therapy, and confirming equivalent doses across statins cannot be performed in our dataset. We do not have available genetic markers to control for these in our analyses, which would improve the robustness of our results. Available quantification of emphysema on imaging would allow us to assess the impact of emphysema on clinical outcomes directly. Likewise, direct measurement of the inflammatory potential of NK cells in the setting of elevated MICA levels would be informative, especially in our COPD sub-analyses. The logical next step for the study would be a multicenter study with thorough clinical and biologic phenotyping with an improved approach to sMICA detection.

## 5. Conclusions

In conclusion, sMICA levels are elevated in those with primary lung cancer compared to those with malignancy metastatic to the lung and no cancer. NSCLC, specifically, is independently associated with the highest levels of peripheral MICA. sMICA levels are also independently associated with the presence of COPD in this cross-sectional analysis. Future studies should focus on well-defined, longitudinal cohorts designed to assess the ability of sMICA to predict disease incidence, progression, and as a marker of response to therapy.

## Figures and Tables

**Table 1:** Demographic, clinical, and soluble MICA*008 description * by the entire study population and lung cancer types.

	Total	No Cancer	NSCLC	SCLC	Cancer in Other Organs	*p*
*n*	567	314	119	9	125	
Age, years	67 (61–71)	67 (60–70)	69 (65–72)	67 (66–69)	68 (64–72)	0.02
Female	138 (24)	72 (23)	38 (32)	3 (33)	20 (16)	0.02
Smoking Current Former Never	161 (28.9) 193 (34.6) 204 (36.6)	68 (22) 78 (24.8) 168 (53.5)	57 (47.9) 53 (44.5) 9 (7.6)	5 (55.6) 4 (44.4) ---	26 (26.8) 22 (22.7) 49 (50.5)	<0.001
Ever Smoker	325 (58)	146 (46.5)	110 (92.4)	9 (100)	48 (38.4)	<0.001
FEV1, L %predicted	1.8 (0.9) 62 (28)	1.9(1.0) 63 (31)	1.9 (0.7) 63 (22)	1.8 (0.5) 56 (21)	1.4 (0.8) 53 (25)	0.02 0.10
FEV1/FVC	0.62 (0.15)	0.62 (0.17)	0.66 (0.12)	0.69 (0.06)	0.59 (0.14)	0.23
RV/TLC	0.53 (0.15)	0.52 (0.15)	0.53 (0.13)	0.49 (0.07)	0.56 (0.1)	0.64
DLco, %predicted	73 (25)	74 (26)	72 (23)	69 (21)	71 (25)	<0.001
Statin Use	74 (20.4)	42 (13)	22 (18.5)	1 (11)	8 (6.4)	<0.001
sMICA, pg/mL ×10^2^	5.20 (27.7)	2.51 (8.97)	15.8 (57.6)	11.6 (32.3)	2.02 (6.93)	<0.001

* Continuous variables are reported as mean with standard deviation (except for age, which is provided as a median and quartile 1 and quartile 3), and categorical variables are represented as counts with percentages. ANOVA is used to compare continuous variables except for age, where a Kruskal–Wallis Test has been performed. Categorical variables are compared via Chi^2^ test. Abbreviations: FEV1 = Forced expiratory volume in one second; FVC = Forced vital capacity; RV = Residual Volume; TLC = Total lung capacity; DLco = Diffusion capacity; sMICA = soluble MICA*008.

**Table 2:** Soluble MICA*008 levels compared across clinically relevant variables *.

Category–Full	Mean (SD) sMICA pg/mL ×10^2^	Range sMICA pg/mL ×10^2^	*p*-Value
**A**. **Smoking Category**			
Never Smoker	2.25 (12.8)	0–166.1	0.07
Former Smoker	5.81 (34.1)	0–424.2	
Current smoker	9.11 (34.2)	0–288.4	
**B**. **Ever Smoker**			
Never Smoker	2.25 (12.8)	0–166.1	0.04
Ever Smoker	7.95 (35.5)	0–424.2	
**C**. **Statin Use**			
No statin use	9.58 (38.4)	0–424.2	0.17
Statin use	3.40 (13.1)	0–97.4	
**D**. **GOLD Stage**			
GOLD I	9.50 (45.4)	0–424.2	0.60
GOLD II	8.67 (39.1)	0–288.4	
GOLD III	3.46 (9.08)	0–50.2	
GOLD IV	6.37 (15.4)	0–56.7	
**E**. **Lung Cancer Stage**			
Stage 1	15.1 (40.4)	0–166.1	0.13
Stage 2	---	---	
Stage 3	7.35 (17.1)	0–71.2	
Stage 4	46.1 (102.0)	0–424.2	
**F**. **Presence of Lung Cancer**			
Primary lung cancer	15.9 (54.9)	0–288.4	<0.001
Rest of population	2.31 (8.32)	0–50.8	
**G**. **Smoking and Lung Cancer**			
Non-smoker	1.77 (.44)	0–56.6	<0.001
Smoker without Lung Cancer	3.11 (9.44)	0–56.6	
Lung cancer	14.9 (54.9)	0–424.2	

* Bivariate comparisons performed using ANOVA for groups of more than two variables; Student’s *t*-test *p*-value is reported. A. Never vs. Former vs. Current Smoker. Former smokers were considered to have not smoked within one month of enrollment. B. Ever Smoker vs. Never Smoker. Ever smoker includes both current and former smokers. C. Reported use of statin therapy vs. not. D. Comparison between GOLD stages. E. Comparison between lung cancer stage in those with primary lung malignancy. F. Primary Lung Cancer vs. Rest of Population. The rest of the population includes those without cancer, those with benign nodules, and those with primary cancer in another organ. G. Comparison between those actively not smoking (Never or Ever Smokers), Current Smokers without lung cancer, and those with lung cancer.

**Table 3:** Multivariate linear regression comparing soluble MICA*008 levels in cancer types vs. those without cancer *.

	sMICA ×10^2^ pg/mL (95% CI)	*p*-Value
Cancer Type		
NSCLC	14.2 (3.57 to 24.9)	0.01
SCLC	7.69 (−1992 to 3531)	0.58
Other Cancer	2.07 (−12.4 to 16.6)	0.77
No Cancer	(ref)	(ref)
FEV1%-predicted	0.07 (−0.12 to 0.25)	0.50
Smoking Category		
Former	2.51 (−11.2 to 16.2)	0.72
Current	2.70 (−10.2 to 15.6)	0.68
Never	(ref)	(ref)
Statin Therapy	−8.34 (−19.7 to 3.01)	0.15

* Controlled for all covariates in the table. Cancer types are compared to those without cancer as the reference value. Current and former smokers are compared to never smokers as the reference value.

**Table 4:** Multivariate linear regression comparing soluble MICA levels in primary lung cancer vs. the rest of the study population *.

	sMICA ×10^2^ pg/mL (95% CI)	*p*-Value
Primary Lung Cancer	12.5 (2.85 to 22.2)	0.011
FEV1%-predicted	0.07 (−0.11 to 0.25)	0.46
Smoking Category		
Former	2.88 (−10.3 to 16.0)	0.67
Current	3.10 (−9.36 to 15.6)	0.62
Never	(ref)	(ref)
Statin Therapy	−8.02 (−19.0 to 3.00)	0.15

* Controlled for all covariates in the table. Current and former smokers are compared to never smokers as the reference value.

**Table 5:** Multivariate linear regression comparing soluble MICA levels in participants with COPD to those without COPD * in the sub-population without lung cancer †.

	sMICA ×10^2^ pg/mL (95% CI)	*p*-Value
COPD	4.38 (0.38 to 8.39)	0.04
Smoking Category		
Former	−0.04 (−5.00 to 3.90)	0.81
Current	2.13 (−2.07 to 6.33)	0.32
Never	(ref)	(ref)
Statin Therapy	−3.47 (−7.75 to 0.08)	0.11

* Controlled for all covariables in the table. † *n* = 439.

## Data Availability

The data used in this study are available upon written request to the senior authors of this manuscript.

## Supplementary Materials

The following supporting information can be downloaded at: https://www.mdpi.com/article/10.3390/jcm15041496/s1. Tables S1–S4: Sensitivity analysis of Tables S1–S4 with the unreadably low levels of sMICA*008 removed. Table S1. Description of cohort for only participants expressing MICA*008; Table S2. Soluble MICA levels compared across clinically relevant variables cohort for only participants expressing MICA*008*; Table S3. Multivariable linear regression comparing soluble MICA levels in cancer types vs. those without cancer cohort for only participants expressing MICA*008*; Table S4. Multivariable linear regression comparing soluble MICA levels in participants with COPD to those without COPD* in the sub-cohort without lung cancer cohort for only participants expressing MICA*008.
