# Supplementary material for: Soluble Major Histocompatibility Complex I-Related Chain A (sMICA)*008 Levels Associate with Smoking, Presence of Chronic Obstructive Pulmonary Disease, and Prevalence of Lung Cancer
Source: J Clin Med. 2026 Feb 14;15(4):1496. doi: 10.3390/jcm15041496 (PMC12942099; doi:10.3390/jcm15041496)
Supplement: Supplementary file 1 [file jcm-15-01496-s001.zip › jcm-3941440-supplementary.pdf]

Supplementary Material: Soluble Major Histocompatibility Complex I-related Chain A (sMICA)*008 Levels Associate with Smoking, Presence of Chronic Obstructive Pulmonary Disease, and Incidence of Lung Cancer.

Robert M. Burkes MD MSCR[1,2], , Mauricio Orozco-Levi MD, PhD, MSc.[3,4,5,6] ,Alba Ramírez-Sarmiento PT, PhD[3,4,5,6], Albert Sánchez-Font MD, PhD[7], Joaquim Gea MD, PhD[7,8,9] , Michael T. Borchers PhD[1,2].

1. Division of Pulmonary, Critical Care and Sleep Medicine, University of Cincinnati, College of Medicine, Cincinnati, USA.
2. Department of Veterans Affairs, Cincinnati, VA Hospital, Cincinnati, USA.
3. Research Center, Fundación Cardiovascular de Colombia. Calle 158 A # 23-58, Floridablanca, Santander (Colombia).
4. Group of Research in Muscle, Training and Lung Diseases (EMICON), Ministry of Science and Technology (MINCIENCIAS), Calle 26 # 57-83, Bogotá, Colombia.
5. Respiratory Department, Hospital Internacional de Colombia, Fundación Cardiovascular de Colombia. Floridablanca, Kilómetro 7 Autopista Piedecuesta, Santander, Colombia.
6. Department of Medicine, Universidad de Santander (UDES), Calle 70 # 55-210, Bucaramanga, Santander, Colombia.
7. Respiratory Medicine Department, Hospital del Mar, Passeig Maritim 25, 08003. Barcelona Spain.
8. Department of Medicine and Life Sciences, Universitat Pompeu Fabra (UPF), Barcelona, Spain.
9. IMIM (Hospital del Mar, Medical Research Institute), Barcelona, Spain.

**Supplementary Table S1. Description of cohort for only participants expressing MICA*008**						
	Total cohort	No Cancer	NSCLC	SCLC	Cancer in other organ	P
n	191	124	31	2	34	
Age, years	66 (59-70)	66 (59-69)	69 (59-74)	45 (35-54)	66 (64-70)	<0.001
Female	46 (24)	33 (26)	6 (20)	1 (2)	6 (18)	0.53
Smoking<br>Current<br>Former<br>Never	 63 (33)<br>82 (43)<br>46 (24)	 31 (25)<br>60 (48)<br>33 (27)	 19 (61)<br>10 (32)<br>2 (7)	 ---<br>2 (100)<br>---	 13 (38)<br>10 (29)<br>11 (33)	 0.002
Ever Smoker	145 (75)	91 (73)	29 (94)	2 (100)	23 (68)	0.06
FEV1, L<br>%predicted	1.6 (1.0)<br>59 (28)	1.7(1.2)<br>54 (31)	2.3 (0.8)<br>72 (17)	2 (0.6)<br>58 (11)	1.4 (0.9)<br>47 (23)	0.01<br>0.04
FEV1/FVC	0.67 (0.11)	0.57 (0.18)	0.68 (0.09)	0.70 (0.7)	0.55 (0.14)	0.004
RV/TLC	0.55 (0.16)	0.56 (0.17)	0.48 (0.13)	0.53 (0.07)	0.57 (0.14)	0.12
DLco, %predicted	76 (23)	74 (24)	83 (17)	84 (22)	73 (24)	0.23
Statin Use	23 (16)	15 (17)	3 (10)	1 (50)	4 (16)	0.46
sMICA, pg/ml	143 (14-915)	31 (11-446)	1225 (347-7122)	5201 (662-9740)	320 (71-568)	<0.001

**Supplementary Table S2. Soluble MICA levels compared across clinically relevant variables cohort for only participants expressing MICA*008***			
**Category -- Full**	**Mean (SD) – MICA pg/ml x10^2**	**Range**	**P-Value**
**A. Smoking Category**			
Never Smoker	2.12 (31.0-11.6)	0.04-16.6	0.07
Former Smoker	0.21 (0.10-3.83)	0-42.4	
Current smoker	4.07 (37.0-38.3)	0-28.8	
**B. Ever Smoker**			
Never Smoker	2.12 (0.31-11.6)	0-16.6	0.04
Ever Smoker	1.18 (0.12-90.0)	0-42.4	
**C. Statin Use**			
No statin use	347 (18-1491)	0-42415	0.17
Statin use	361 (109-799)	0-9740	
**D. GOLD Stage**			
GOLD I	0.71 (10-11.7)	0-42415	0.60
GOLD II	1.80 (11-11.6)	0-28840	
GOLD III	2.79 (0.14-6.81)	0-5018	
GOLD IV	1.07 (0.17-14.9)	0-5664	
**E. Lung Cancer Stage**			
Lung cancer stage 1	26.5 (3.70-31.2)	0-16.61	0.13
Stage 2	---	---	
Stage 3	2582 (749-3980)	0-71.2	
Stage 4	15.7 (84.5-20.8)	0-42.4	
**F. Presence of Lung Cancer**			
Primary lung cancer	12.3 (3.47-71.2)	0-42.4	<0.001
Rest of cohort	0.76 (0.12-5.33)	0-56.6	
**G. Smoking and Lung Cancer**			
Non-smoker	0.20 (0.11-2.79)	0-56.6	<0.001
Smoker without Lung Cancer	3.34 (24-12.0)	0-56.6	
Lung cancer	12.3 (3.47-71.2)	0-42.4	

*** Bivariable comparisons performed using ANOVA for groups of more-than-two variables, otherwise Student's T-test P-value is reported.**
A. Never vs. Former vs. Current Smoker
B. Ever Smoker vs Never Smoker
C. Reported use of statin therapy vs. not
D. Comparison between GOLD stages.
E. Comparison between lung cancer stage in those with primary lung malignancy
F. Primary Lung Cancer vs. Rest of Cohort
G. Comparison between those actively not smoking (Never or Ever Smokers), Current

Smokers without lung cancer, and those with lung cancer

Supplementary Table S3. Multivariable linear regression comparing soluble MICA levels in cancer types vs. those without cancer cohort for only participants expressing MICA*008*		
	**sMICA pg/ml x10^2 (95% CI)**	**P**
Cancer Type		
NSCLC	53.6 (26.9 to 80.3)	<0.001
Other Cancer	2.24 (-28.6 to 28.2)	0.86
No Cancer	(ref)	(ref)
FEV1 %-predicted	.07 (-0.36 to 0.50)	0.76
Smoking Category		
Former	0.48 (-26.1 to 27.0)	0.97
Current	-1.22 (-27.4 to 30.0)	0.93
Never	(ref)	(ref)
Statin Therapy	-13.4 (-39.3 to 12.6)	0.31
***Controlled for all covariables in the table**		

Supplementary Table S4. Multivariable linear regression comparing soluble MICA levels in participants with COPD to those without COPD* in the sub-cohort without lung cancer cohort for only participants expressing MICA*008		
	**sMICA pg/ml x10^2 (95% CI)**	**P**
COPD	32.2 (15.9 to 48.6)	<0.001
Smoking Category		
Former	-1.06 (-27.7 to 25.6)	0.94
Current	313 (-23.4 to 29.7)	0.82
Never	(ref)	(ref)
Statin Therapy	-10.9 (-37.2 to 15.3)	0.41
***Controlled for all covariables in the table**		
